# Ocular Biometric Characteristics of Chinese with History of Acute Angle Closure

**DOI:** 10.1155/2018/5835791

**Published:** 2018-10-17

**Authors:** Wei-ran Niu, Chun-qiong Dong, Xi Zhang, Yi-fan Feng, Fei Yuan

**Affiliations:** Department of Ophthalmology, Zhongshan Hospital of Fudan University, Shanghai 200032, China

## Abstract

**Purpose:**

To investigate the biometric characteristics of Chinese patients with a history of acute angle closure (AAC).

**Methods:**

In this clinic-based, retrospective, observational, cross-sectional study, biometric parameters of eyes were acquired from a general population of Chinese adults. The crowding value (defined as lens thickness (LT); central corneal thickness (CCT); anterior chamber depth (ACD)/axial length (AL)) was calculated for each patient. Logistic regression analysis was performed to identify risk factors for AAC. Receiver operating characteristic (ROC) curves were plotted, and biometric variables were compared to compile a risk assessment for AAC.

**Result:**

This study included 1500 healthy subjects (2624 eyes, mean age of 66.54 ± 15.82 years) and 107 subjects with AAC (202 eyes, mean age of 70.01 ± 11.05 years). Eyes with AAC had thicker lens (*P* ≤ 0.001), shallower anterior chamber depth (*P* ≤ 0.001), and shorter axial length (*P* ≤ 0.001) than healthy eyes. Logistic regression analysis and ROC curve analysis indicated that a crowding value above 0.13 was a significant (*P* < 0.05) risk factor for the development of AAC.

**Conclusions:**

Biometric parameters were significantly different between the eyes from the AAC group to the normal group. Ocular crowding value might be a new noncontact screening method to assess the risk of AAC in adults.

## 1. Introduction

Glaucoma is a leading cause of ocular morbidity and blindness worldwide [1]. It is estimated that by 2020, there will be 79.6 million people suffering from glaucoma, of which 26% will have primary angle closure glaucoma (PACG) [2]. Previous studies have stated that PACG is responsible for nearly half the cases of glaucoma-related blindness in the world, and the prevalence of this condition is highest in China [2, 3].

In the Primary Angle Closure Preferred Practice Pattern® (PPP) guidelines (2016), acute angle closure crisis (AACC) is described as a suddenly occluded angle with symptomatic high IOP [4]. Acute angle closure (AAC) can occur rapidly, recur, and cause permanent vision loss or blindness [5–7]. Eyes with optic neuropathy caused by AAC will be diagnosed as primary angle-closure glaucoma. Since approximately half of fellow eyes of acute angle-closure patients can develop AACCs within 5 years, the fellow eye is also at high risk of AAC [4]. Preventive interventions can be effective in the treatment of patients with AAC [8, 9]; managing AACC successfully has been one of the main clinic objectives of PAC and, it is of paramount importance to assess the risk of AAC properly.

Notably, many ways have been used for detecting a closed angle to diagnose primary angle closure disease (PACD) instead of assessing the risk of AAC. For example, gonioscopy examination is the current gold standard for the detection of PACD [10], and is not so suitable for case-finding or large-scale population screening; Van Herick's method has been used as a substitutive assessment method of gonioscopy [11, 12], screening results of which may vary from one ophthalmologist to another [13].

Knowledge of biometric parameters is essential for understanding the development of ocular growth and AAC pathologies. Some ocular anatomical characteristics such as short axial length and shallow anterior chamber depth have been reported to be major risk factors of AAC [14–18], and in other words, the “small eyes” are at a higher risk of developing AAC. However, the traditional biometric parameters such as anterior chamber depth or lens vault are not strong predictors of ACC [19–21]. We speculate that the crowding condition of the eye would be a more important factor to trigger an AAC, and thus the parameters describing the condition should be more appropriate predictors for AAC.

It remains difficult to investigate the in-depth pathologies of AAC; but there may be a way to assess the risk of it. With identification of high-risk individuals, the development of AAC could be interrupted at the right time. Towards this end, we collected the ocular biometric parameters of Chinese subjects with an AAC history and compared those of AAC eyes to healthy eyes to identify a new method to assess the risk of AAC.

## 2. Materials and Methods

This retrospective, observational, cross-sectional study was approved by the Department of Ophthalmology, Zhongshan Hospital, Fudan University, Shanghai, China, and conducted in accordance with the Declaration of Helsinki.

### 2.1. Study Population

Here, 1810 subjects were recruited consecutively between October 2013 and April 2015. The subjects were either outpatients or inpatients in the Department of Ophthalmology, Zhongshan Hospital, Fudan University, Shanghai, China. All subjects were over 18 years old and from the ethnic Chinese Han population. Both healthy eyes and those with a history of AAC were selected; both eyes of each patient were included in the study. The healthy group included patients who presented at our clinic for glasses, minor external ocular discomfort, or cataracts with normal angles and optic nerve head. Patients with an AAC history should be clinic silent and intraocular pressures (IOPs) should be maintained in a normal range.

Data from subjects with AAC who were surgically treated for glaucoma or had a laser treatment such as laser peripheral iridotomy (LPI) or were in the acute stage, with a history of ocular surgery, trauma, tumor, and pathologies such as detachment of retina or second glaucoma were excluded. Subjects younger than 18 years were also excluded. Pilocarpine treatment was discontinued at least one day before the examination, and IOP were measured during the examining period.

### 2.2. Study Design

All subjects underwent a thorough ophthalmic examination, which included slit-lamp biomicroscopy, IOP measurement by applanation tonometry, fundus examination, and measurements of other ocular biometrics. Central corneal thickness (CCT), lens thickness (LT), anterior chamber depth (ACD), and axial length (AL) were measured using a LENSTAR LS 900 (Haag-Streit, Koeniz, Switzerland). The associated measurements were carried out by the same investigator, and the LENSTAR LS 900 measurement procedure has previously been described in detail elsewhere [22].

### 2.3. Statistical Analysis

All statistical analyses were performed using SPSS (Windows ver. 20.0; SPSS Inc., Chicago, IL, USA). Basic descriptive statistics were calculated on all data reported as mean value ± standard deviation. Categorical data were compared using the chi-squared test, and numerical data were compared employing one-way ANOVA and Student's *t*-test. Numerical data of eyes from one subject were compared using the paired sample *t*-test. All tests were two-tailed, and *P* values were considered statistically significant at *P* < 0.05.

Biometric parameters with statistically significant differences between the study group and the control group were used to build a binary conditional logistic regression analysis model to assess the risk of AAC. Receiver operating characteristic (ROC) curves were plotted using crowding values (defined in Results) to obtain a suitable cutoff value to separate healthy from eyes at risk of AAC. The best sensitivity/specificity relationship was determined using the cutoff point extrapolated from the area under ROC curves and predicted probabilities.

## 3. Results

### 3.1. The Demographic Characteristics of all Subjects

Complete data were available for 107 subjects with an AAC history (202 eyes) and 1500 subjects (2624 eyes) in the control group (Table 1). The IOP of all the subjects were in the normal range from 10.0 to 21.0 mmHg.

### 3.2. Differences between the Biometric Parameters in the AAC and Control Groups and between Right and Left Eyes

AAC cases were significantly older (70 ± 11 years) than the control group (67 ± 16 years) (*P*=0.026). There were statistically more females in the AAC group compared with the control group (*P*=0.021), and there were no significant differences in all four biometric parameters between the right and left eyes of the AAC group (Table 2). The CCT, LT, and AL of two groups were significantly different (Table 3).

### 3.3. Correlation between AAC Biometric Parameters Based on Logistic Regression Analysis

Shallower ACD and shorter AL as well as LT were significantly associated with the prediction of AAC by binary conditional logistic regression analysis, after adjustment for age and sex (Table 4). After adjusting for all other parameters, older age (ORs 1.018; *P* < 0.0001) was shown to be significantly associated with AAC by conditional logistic regression analysis.

### 3.4. Crowding Value and Receiver Operating Characteristic Curves

A crowding value was calculated from the following equation which was created based on our results:

(1) $$\begin{array}{c} \text{crowding value}=\frac{\left(\text{CCT}+\text{LT}-\text{ACD}\right)}{\text{AL}}. \end{array}$$

**Figure 1:**
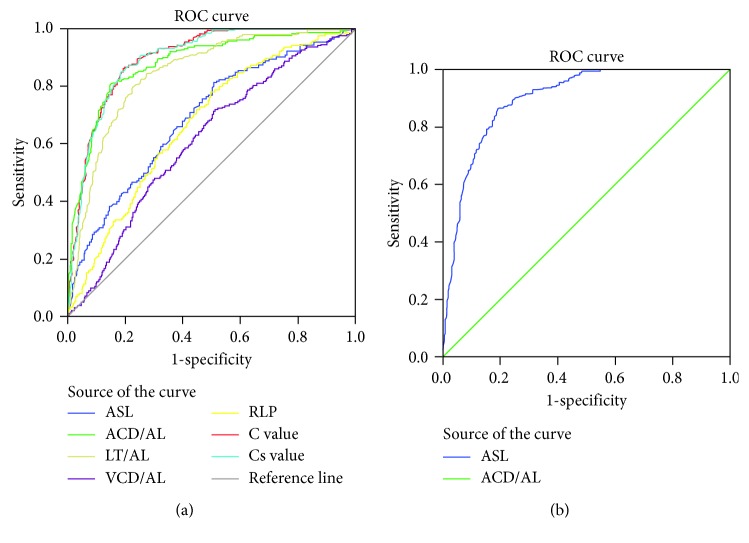
ROC curves plotting sensitivity against one-specificity (Az ROC: area under the ROC curve). In our study, a cutoff of 0.13 for crowding value seems to be the best value to separate healthy eyes from those at risk of AAC.

ROC curves were then plotted using crowding values to assess the risk of AAC. ROC curve analysis showed that the optimal probability cutoff for the assessment of AAC was a crowding value over 0.13, with the area under the curve being 0.899 ± 0.009 (Figure 1). The corresponding sensitivity and specificity of crowding measurement were 86.6% and 80.6%, respectively. ROC curves using other formulas previously reported in the literature to determine the risk of angle closure were also plotted (Table 5). Results of simple crowding value (calculated as (LT − ACD)/AL) are also listed in Table 5.

## 4. Discussion

The Asian population has a high prevalence of AAC [2, 3, 23, 24]. Although AAC is well-studied, the pathological processes of AAC remains poorly understood. In the present study, we investigate the biometric characteristic of Han Chinese subjects with a history of AAC.

In many studies, only biometric data from one eye, commonly the right eye, were measured [19, 25]. PACD is a bilateral disease. Although 90% of AACs are unilateral, approximately half of fellow eyes of acute angle-closure patients can develop AACCs within 5 years; therefore, the contralateral eye of patients with a monocular AAC would be at high risk for AAC [18, 26, 27]. For this reason, biometric data from contralateral eyes were important for this analysis; accordingly data from both eyes were collected and analyzed.

There was no significant difference between the biometric parameters of the right eye and the left eye for patients in the AAC group, a finding which disagrees with previous study [28]. We presume that there might be two reasons for this contradictory finding. Firstly, the data from patients in the acute stage of AAC were excluded from this study, and data from patients in poor condition who underwent surgery or LPI were also excluded. Removal of these confounding factors led to a more homogenous AAC group.

In the present study, there were more females and older subjects in the AAC group in agreement with previous research [3, 29]. We found that the eyes of the AAC group had a shorter AL, shallower ACD, and thicker LT than normal eyes, consistent with other publications [15,30–32]. There was no difference in CCT between the 2 groups; however, in our opinion, it would be too thin a cornea for eyes with a short AL in AAC group.

Earlier studies failed to identify an eye with AAC simply by the value of ACD [20, 27], LT [20,33–35], AL [21,36–38], and CCT [19, 39]. It was reported that patients with an ACD < 2.55 mm and a LT > 4.66 mm were at higher risk of APAC, with sensitivity of 60% and 67.6% and specificity of 65.3% and 60.5%, respectively [40].

Formulas were used to assess the risk of angle closure such as anterior segment length (ASL: summation of CCT, ACD, and LT) [19], the contribution of individual ocular components to the total axial (ACD/AL, LT/AL, VCD vitreous chamber depth/AL) [41], and the relative lens position (RLP: calculated as (ACD + 1/2LT)/AL) [42].

In the present study, we calculated a crowding value as follows: (LT+CCT-ACD)/AL according to the results of the logistic regression model. All four biometric parameters were shown to play a role in the development of AAC.

All these variables were calculated based on the recorded results of biometric parameters in the present study. The crowding value had the highest AUROC which means it is more sensitive and more specific than all the other variables mentioned in previous studies to assess the risk of AAC [40]. Changes in eyes with age such as an increase in LT make the structure of eyes more crowded, and a record of crowding value may help us to understand the development of ocular growth and AAC pathologies.

The results of our study should be interpreted with some limitations in mind. Firstly, because all patients were of Han Chinese descent and were recruited from the Department of Ophthalmology, Zhongshan Hospital, the results of this study may not be applicable to other racial groups and may not be generalizable to the larger population. Secondly, some studies have shown that cortical or nuclear cataracts may also be associated with angle closure [41]. However, the presence of severe cataracts makes measurements of LT and AL difficult, so such patients were excluded. Thirdly, because not all patients should be examined with gonioscopy, PACS eyes without any complaints, special biometric parameters, and medical history might be included in the healthy participants although studies suggest that the majority of patients with PACS will not develop either PAC or PACG [8, 43]. Lastly, we excluded patients who had accepted laser peripheral iridotomy treatment or surgery as this might have led to unnatural biometric differences between the right and left eyes.

## 5. Conclusions

In conclusion, ACC eyes have higher crowding values in terms of biometric parameters. Determination of ocular crowding value using ocular biometric parameters may represent a novel and rapid method to assess the risk of AAC. Future studies with a larger population representing different ethnic groups are needed to test the reliability and repeatability of our findings.

## Figures and Tables

**Table 1:** Demographic characteristics of subject groups.

Parameter	AAC group (*n* = 107)	Control group (*n* =1500)	*P* value
Female sex, *n* (%)	72 (67.3)	837 (55.8)	0.021
Mean age ± SD (y)	70.0 ± 11.1	66.5 ± 15.8	0.026
Right eye, *n* (%)	105 (52.0)	1411 (53.8)	0.661

**Table 2:** Comparison of biometric parameters between right and left eyes in the AAC and the control groups.

	AAC group				Control group			
	Right eye (mean ± SD)	Left eye (mean ± SD)	*N* (right/left)	*P* value (paired Sample t- test)	Right eye	Left eye	*N* (right/left)	*P* value (paired Sample *t*-test)
CCT, μm	544.93 ± 41.82	539.89 ±35.80	87/87	0.155	538.03 ± 34.87	539.72 ± 36.05	1112/1112	0.000
AD, mm	1.82 ± 0.32	1.86 ± 0.39	87/87	0.274	2.69 ± 0.51	2.67 ± 0.51	1112/1112	0.134
LT, mm	4.89 ± 0.42	4.89 ± 0.42	87/87	0.915	4.37 ± 0.47	4.38 ± 0.48	1112/1112	0.050
AL, mm	22.89 ± 1.45	22.94 ± 1.61	87/87	0.336	24.80 ± 2.44	24.70 ± 2.37	1112/1112	0.000

CCT = central corneal thickness; ACD = anterior chamber depth; LT = lens thickness; AL = axial length.

**Table 3:** Comparison of biometric parameters between the AAC and the control groups.

	AAC (mean ± SD)	Control group (mean ± SD)	*P* value (Student's *t*-test)
CCT, *μ*m	544.25 ± 38.97	539.17 ± 36.02	0.055
ACD, mm	1.85 ± 0.37	2.65 ± 0.50	0.000
LT, mm	4.88 ± 0.41	4.40 ± 0.47	0.000
AL, mm	22.88 ± 1.45	24.70 ± 2.40	0.000

CCT = central corneal thickness; ACD = anterior chamber depth; LT = lens thickness; AL = axial length.

**Table 4:** Results of binary logistic regression analysis of biometric parameters for the prediction of AAC.

	Adjusted odds ratios	*P* value	95% confidence interval
CCT	1.005	0.077	1.000–1.009
ACD	0.014	0.000	0.009–0.031
LT	1.796	0.065	0.974–2.351
AL	0.872	0.017	0.745–0.972

CCT = central corneal thickness; ACD = anterior chamber depth; LT = lens thickness; AL = axial length.

**Table 5:** Area under the receiver operating characteristic curve (AUROC), sensitivity, specificity, and cutoff value in healthy and AAC Subjects.

	AUROC	Sensitivity, specificity	Cutoff
ASL, mm	0.690	48.5%, 81.7%	≤7.58
Ratio (ACD/AL)	0.879	84.8%, 81.2%	≤0.09
Ratio (LT/AL)	0.845	85.5%, 77.1%	≥0.20
Ratio (VCD/AL)	0.669	72.0%, 42.7%	≤0.89
Ratio (RLP)	0.611	50.2%, 78.2%	≤0.19
Ratio (crowding value)	0.899	86.6%, 80.6%	≥0.13
Ratio (simple crowding value)	0.897	84.7%, 81.8%	≥0.11

ACD = anterior chamber depth; LT = lens thickness; AL = axial length; ASL = anterior segment length; RLP = relative lens position; simple crowding value = (LT - ACD)/AL. *P* < 0.05 was considered statistically significant.

## Data Availability

The datasets used to support this study are provided as a supplementary material file which includes the records of the demographic characteristics and biometric parameters of all the subjects.

## Supplementary Materials

Supplementary Materials The supplementary material file includes the records of the demographic characteristics and biometric parameters of all the subjects.
